# Supplementary material for: Multi‐dataset identification of innovative feature genes and molecular mechanisms in keratoconus
Source: J Cell Mol Med. 2024 Sep 19;28(18):e70079. doi: 10.1111/jcmm.70079 (PMC11412914; doi:10.1111/jcmm.70079)
Supplement: Supplementary file 2 — Material S2. [file JCMM-28-e70079-s002.zip › 7.GSEA analysis/1.ARL11/KEGG_HUNTINGTONS_DISEASE.html]

Details for gene set KEGG\_HUNTINGTONS\_DISEASE[GSEA]

|  || Dataset | ARL11.group.cls#h\_versus\_l |
| Phenotype | group.cls#h\_versus\_l |
| Upregulated in class | l |
| GeneSet | KEGG\_HUNTINGTONS\_DISEASE |
| Enrichment Score (ES) | -0.20516783 |
| Normalized Enrichment Score (NES) | -1.1576711 |
| Nominal p-value | 0.26868686 |
| FDR q-value | 1.0 |
| FWER p-Value | 0.99 |
Table: GSEA Results Summary

  

Fig 1: Enrichment plot: KEGG\_HUNTINGTONS\_DISEASE      
 Profile of the Running ES Score & Positions of GeneSet Members on the Rank Ordered List

  

| SYMBOL | TITLE | RANK IN GENE LIST | RANK METRIC SCORE | RUNNING ES | CORE ENRICHMENT || 1 | PLCB2 | na | 134 | 0.619 | 0.0593 | No |
| 2 | GRM5 | na | 2391 | 0.271 | 0.0216 | No |
| 3 | DNAI1 | na | 2624 | 0.259 | 0.0413 | No |
| 4 | TBPL2 | na | 3636 | 0.214 | 0.0338 | No |
| 5 | PPARGC1A | na | 4007 | 0.200 | 0.0436 | No |
| 6 | CLTC | na | 5003 | 0.166 | 0.0317 | No |
| 7 | SLC25A31 | na | 5206 | 0.160 | 0.0422 | No |
| 8 | APAF1 | na | 5299 | 0.157 | 0.0556 | No |
| 9 | HTT | na | 6023 | 0.137 | 0.0486 | No |
| 10 | CASP8 | na | 6150 | 0.133 | 0.0585 | No |
| 11 | HIP1 | na | 6428 | 0.126 | 0.0634 | No |
| 12 | CREBBP | na | 7701 | 0.098 | 0.0366 | No |
| 13 | NDUFA4L2 | na | 8377 | 0.085 | 0.0257 | No |
| 14 | DNAH2 | na | 9239 | 0.071 | 0.0081 | No |
| 15 | AP2A2 | na | 9356 | 0.070 | 0.0118 | No |
| 16 | TP53 | na | 9447 | 0.068 | 0.0162 | No |
| 17 | CASP3 | na | 9607 | 0.066 | 0.0183 | No |
| 18 | DNALI1 | na | 9947 | 0.061 | 0.0147 | No |
| 19 | ITPR1 | na | 10204 | 0.058 | 0.0131 | No |
| 20 | PLCB3 | na | 10228 | 0.057 | 0.0183 | No |
| 21 | NDUFC2 | na | 10744 | 0.051 | 0.0086 | No |
| 22 | DNAH1 | na | 10770 | 0.050 | 0.0130 | No |
| 23 | NDUFS1 | na | 10818 | 0.050 | 0.0167 | No |
| 24 | SP1 | na | 11109 | 0.046 | 0.0130 | No |
| 25 | POLR2A | na | 11674 | 0.039 | 0.0007 | No |
| 26 | REST | na | 12616 | 0.030 | -0.0234 | No |
| 27 | HDAC1 | na | 12805 | 0.029 | -0.0259 | No |
| 28 | CREB3L1 | na | 12911 | 0.028 | -0.0261 | No |
| 29 | COX7A2L | na | 12931 | 0.028 | -0.0239 | No |
| 30 | POLR2J2 | na | 12965 | 0.027 | -0.0220 | No |
| 31 | TAF4 | na | 13402 | 0.024 | -0.0322 | No |
| 32 | COX6B2 | na | 13815 | 0.020 | -0.0421 | No |
| 33 | NDUFB5 | na | 13830 | 0.020 | -0.0404 | No |
| 34 | NDUFV3 | na | 13986 | 0.019 | -0.0430 | No |
| 35 | GPX1 | na | 14044 | 0.018 | -0.0428 | No |
| 36 | AP2M1 | na | 14127 | 0.018 | -0.0433 | No |
| 37 | NDUFA1 | na | 14213 | 0.017 | -0.0440 | No |
| 38 | NDUFV1 | na | 14593 | 0.014 | -0.0535 | No |
| 39 | DCTN1 | na | 14901 | 0.012 | -0.0612 | No |
| 40 | UQCRQ | na | 15016 | 0.011 | -0.0634 | No |
| 41 | NDUFS8 | na | 15042 | 0.011 | -0.0630 | No |
| 42 | CLTA | na | 15063 | 0.011 | -0.0625 | No |
| 43 | NDUFB1 | na | 15196 | 0.010 | -0.0653 | No |
| 44 | NDUFS6 | na | 15253 | 0.009 | -0.0660 | No |
| 45 | POLR2C | na | 15275 | 0.009 | -0.0656 | No |
| 46 | NDUFC1 | na | 15395 | 0.008 | -0.0682 | No |
| 47 | COX6B1 | na | 15603 | 0.007 | -0.0735 | No |
| 48 | NDUFS5 | na | 15692 | 0.006 | -0.0755 | No |
| 49 | SLC25A5 | na | 15717 | 0.006 | -0.0756 | No |
| 50 | SDHC | na | 15742 | 0.005 | -0.0757 | No |
| 51 | TBP | na | 15760 | 0.005 | -0.0757 | No |
| 52 | NDUFB8 | na | 15934 | 0.004 | -0.0802 | No |
| 53 | NDUFA10 | na | 15939 | 0.004 | -0.0799 | No |
| 54 | NDUFA5 | na | 16219 | 0.002 | -0.0878 | No |
| 55 | TBPL1 | na | 16236 | 0.002 | -0.0880 | No |
| 56 | COX8A | na | 16384 | 0.001 | -0.0922 | No |
| 57 | UQCRC1 | na | 16414 | 0.001 | -0.0929 | No |
| 58 | NDUFA3 | na | 16449 | 0.001 | -0.0939 | No |
| 59 | NDUFS4 | na | 16514 | 0.000 | -0.0957 | No |
| 60 | NDUFS3 | na | 16549 | -0.000 | -0.0967 | No |
| 61 | POLR2I | na | 16645 | -0.001 | -0.0993 | No |
| 62 | DCTN2 | na | 16656 | -0.001 | -0.0995 | No |
| 63 | NDUFB10 | na | 16676 | -0.001 | -0.0999 | No |
| 64 | COX7B | na | 16679 | -0.001 | -0.0999 | No |
| 65 | NDUFAB1 | na | 16740 | -0.002 | -0.1015 | No |
| 66 | POLR2G | na | 16746 | -0.002 | -0.1014 | No |
| 67 | AP2S1 | na | 16930 | -0.003 | -0.1065 | No |
| 68 | NDUFA7 | na | 16940 | -0.003 | -0.1064 | No |
| 69 | NDUFB3 | na | 17132 | -0.004 | -0.1115 | No |
| 70 | NDUFB9 | na | 17155 | -0.004 | -0.1117 | No |
| 71 | COX5B | na | 17270 | -0.005 | -0.1145 | No |
| 72 | CREB3L4 | na | 17310 | -0.005 | -0.1151 | No |
| 73 | UQCRC2 | na | 17359 | -0.006 | -0.1159 | No |
| 74 | UQCR11 | na | 17430 | -0.006 | -0.1173 | No |
| 75 | NDUFA8 | na | 17455 | -0.006 | -0.1173 | No |
| 76 | COX7C | na | 17486 | -0.007 | -0.1175 | No |
| 77 | RCOR1 | na | 17538 | -0.007 | -0.1182 | No |
| 78 | NDUFB4 | na | 17540 | -0.007 | -0.1176 | No |
| 79 | BAX | na | 17601 | -0.008 | -0.1185 | No |
| 80 | POLR2H | na | 17645 | -0.008 | -0.1190 | No |
| 81 | CYCS | na | 17719 | -0.008 | -0.1202 | No |
| 82 | DCTN4 | na | 17746 | -0.009 | -0.1201 | No |
| 83 | GNAQ | na | 17828 | -0.009 | -0.1215 | No |
| 84 | POLR2B | na | 17954 | -0.010 | -0.1241 | No |
| 85 | POLR2E | na | 17957 | -0.010 | -0.1232 | No |
| 86 | COX7B2 | na | 18020 | -0.010 | -0.1239 | No |
| 87 | COX7A2 | na | 18098 | -0.011 | -0.1250 | No |
| 88 | NDUFB7 | na | 18113 | -0.011 | -0.1243 | No |
| 89 | PLCB4 | na | 18133 | -0.011 | -0.1237 | No |
| 90 | POLR2J | na | 18190 | -0.012 | -0.1241 | No |
| 91 | CLTB | na | 18193 | -0.012 | -0.1230 | No |
| 92 | TFAM | na | 18238 | -0.012 | -0.1230 | No |
| 93 | POLR2J3 | na | 18355 | -0.013 | -0.1251 | No |
| 94 | PPID | na | 18689 | -0.015 | -0.1332 | No |
| 95 | POLR2L | na | 19007 | -0.017 | -0.1407 | No |
| 96 | SOD1 | na | 19015 | -0.017 | -0.1391 | No |
| 97 | COX6C | na | 19016 | -0.017 | -0.1374 | No |
| 98 | COX6A1 | na | 19083 | -0.017 | -0.1375 | No |
| 99 | VDAC3 | na | 19110 | -0.018 | -0.1365 | No |
| 100 | NDUFS7 | na | 19230 | -0.019 | -0.1380 | No |
| 101 | SDHA | na | 19462 | -0.020 | -0.1427 | No |
| 102 | UQCRH | na | 19528 | -0.021 | -0.1424 | No |
| 103 | NDUFA2 | na | 19631 | -0.021 | -0.1432 | No |
| 104 | NDUFB2 | na | 19632 | -0.021 | -0.1410 | No |
| 105 | POLR2F | na | 19641 | -0.021 | -0.1391 | No |
| 106 | SLC25A6 | na | 19656 | -0.021 | -0.1373 | No |
| 107 | NDUFA6 | na | 19673 | -0.022 | -0.1356 | No |
| 108 | NDUFA9 | na | 20241 | -0.025 | -0.1494 | No |
| 109 | COX4I1 | na | 20260 | -0.026 | -0.1473 | No |
| 110 | CASP9 | na | 20358 | -0.026 | -0.1474 | No |
| 111 | UQCRFS1 | na | 20848 | -0.030 | -0.1584 | No |
| 112 | CREB3 | na | 20880 | -0.030 | -0.1563 | No |
| 113 | POLR2D | na | 21004 | -0.031 | -0.1566 | No |
| 114 | UQCR10 | na | 21044 | -0.032 | -0.1545 | No |
| 115 | UCP1 | na | 21225 | -0.033 | -0.1564 | No |
| 116 | COX5A | na | 21246 | -0.033 | -0.1536 | No |
| 117 | SIN3A | na | 21458 | -0.035 | -0.1561 | No |
| 118 | VDAC2 | na | 21482 | -0.035 | -0.1532 | No |
| 119 | DNAL4 | na | 21573 | -0.036 | -0.1521 | No |
| 120 | HDAC2 | na | 21663 | -0.037 | -0.1510 | No |
| 121 | NDUFV2 | na | 21789 | -0.038 | -0.1508 | No |
| 122 | SDHD | na | 22056 | -0.040 | -0.1544 | No |
| 123 | NDUFS2 | na | 22196 | -0.042 | -0.1541 | No |
| 124 | UQCRB | na | 22209 | -0.042 | -0.1502 | No |
| 125 | CYC1 | na | 22402 | -0.044 | -0.1512 | No |
| 126 | VDAC1 | na | 22460 | -0.045 | -0.1483 | No |
| 127 | NDUFA4 | na | 22537 | -0.045 | -0.1459 | No |
| 128 | CREB3L2 | na | 23209 | -0.052 | -0.1600 | No |
| 129 | SOD2 | na | 23532 | -0.056 | -0.1636 | No |
| 130 | SDHB | na | 23559 | -0.057 | -0.1585 | No |
| 131 | BBC3 | na | 23628 | -0.058 | -0.1546 | No |
| 132 | NDUFB6 | na | 23631 | -0.058 | -0.1488 | No |
| 133 | POLR2K | na | 23784 | -0.060 | -0.1471 | No |
| 134 | AP2B1 | na | 23828 | -0.060 | -0.1422 | No |
| 135 | EP300 | na | 23862 | -0.060 | -0.1370 | No |
| 136 | CLTCL1 | na | 25284 | -0.080 | -0.1700 | No |
| 137 | DLG4 | na | 25739 | -0.086 | -0.1743 | No |
| 138 | COX4I2 | na | 26565 | -0.100 | -0.1880 | No |
| 139 | SLC25A4 | na | 27159 | -0.112 | -0.1938 | Yes |
| 140 | CREB1 | na | 27227 | -0.113 | -0.1842 | Yes |
| 141 | GRIN1 | na | 27606 | -0.121 | -0.1828 | Yes |
| 142 | CREB3L3 | na | 27885 | -0.128 | -0.1777 | Yes |
| 143 | AP2A1 | na | 28060 | -0.132 | -0.1693 | Yes |
| 144 | GRIN2B | na | 28410 | -0.141 | -0.1650 | Yes |
| 145 | COX7A1 | na | 28636 | -0.147 | -0.1564 | Yes |
| 146 | PPARG | na | 28947 | -0.155 | -0.1496 | Yes |
| 147 | HAP1 | na | 29033 | -0.157 | -0.1360 | Yes |
| 148 | DNAL1 | na | 29312 | -0.166 | -0.1271 | Yes |
| 149 | NRF1 | na | 29801 | -0.181 | -0.1227 | Yes |
| 150 | COX6A2 | na | 30087 | -0.190 | -0.1115 | Yes |
| 151 | BDNF | na | 30179 | -0.194 | -0.0944 | Yes |
| 152 | TAF4B | na | 30977 | -0.220 | -0.0951 | Yes |
| 153 | DNAI2 | na | 31307 | -0.232 | -0.0809 | Yes |
| 154 | PLCB1 | na | 32251 | -0.272 | -0.0804 | Yes |
| 155 | TGM2 | na | 32693 | -0.300 | -0.0626 | Yes |
| 156 | IFT57 | na | 33316 | -0.345 | -0.0454 | Yes |
| 157 | COX8C | na | 33951 | -0.412 | -0.0217 | Yes |
| 158 | CREB5 | na | 34086 | -0.433 | 0.0186 | Yes |
Table: GSEA details [plain text format]

  

Fig 2: KEGG\_HUNTINGTONS\_DISEASE      
 Blue-Pink O' Gram in the Space of the Analyzed GeneSet

  

Fig 3: KEGG\_HUNTINGTONS\_DISEASE: Random ES distribution      
 Gene set null distribution of ES for **KEGG\_HUNTINGTONS\_DISEASE**
